# Healthy and moribund Zhikong scallops (*Chlamys farreri*) developed different viral communities during a mass mortality event

**DOI:** 10.1128/msystems.00342-25

**Published:** 2025-05-14

**Authors:** Jiaxiang Li, Kaiyang Zheng, Wei Ding, Longfei Lu, Yantao Liang, Yao Xiong, Zhongcheng Wei, Chen Gao, Yue Su, Ziyue Wang, Xin Chen, Zhenmin Bao, Xiaoli Hu, Andrew McMinn, Min Wang

**Affiliations:** 1 MoE Key Laboratory of Evolution and Marine Biodiversity, College of Marine Life Sciences, Institute of Evolution and Marine Biodiversity, Frontiers Science Center for Deep Ocean Multispheres and Earth System, and Center for Ocean Carbon Neutrality, Ocean University of China 12591, Qingdao, Shandong, China; 2 Key Laboratory of Biological Genetics and Breeding, Ministry of Education, Ocean University of China 12591, Qingdao, Shandong, China; 3 Fourth Institute of Oceanography, Ministry of Natural Resources 635955 , Beihai, Guangxi, China; 4 Weihai Changqing Ocean Science Technology Co., Ltd., Weihai, China; 5 UMT-OUC Joint Centre for Marine Studies, Qingdao, China; 6 Haide College, Ocean University of China 12591, Qingdao, Shandong, China; 7 Institute for Marine and Antarctic Studies, University of Tasmania 445071 , Hobart, Tasmania, Australia; 8 The Affiliated Hospital of Qingdao University 235960, Qingdao, Shandong, China; The University of Maine, Orono, Maine, USA

**Keywords:** *Chlamys farreri*, metagenome, viral assemblages, mortality, auxiliary metabolic genes

## Abstract

Viral assemblages of scallops are still relatively unknown. Here, metagenomic analysis was used to study virus communities in the gut of scallops to establish the first scallop virome data set (SVD); this contains 7,447 viral operational taxonomic units. Protein-sharing networks and phylogenetic analyses demonstrated the high diversity and novelty of the SVD, which is very different from viromes from other habitats. Potentially pathogenic viruses are prevalent in the gut of scallops. In particular, the novel smacoviruses were identified, indicating that scallops may be a potential hotspot for this viral group. Inference of virus-host associations found extensive interactions between viruses and major prokaryotic lineages. Intriguingly, moribund scallops showed a higher diversity of auxiliary metabolic genes (AMGs) related to amino acid metabolism and cofactor and vitamin genes, while healthy scallops had fewer AMGs, with those present focusing on secondary metabolite biosynthesis and carbohydrate metabolism. These findings provide the first landscape of scallop gut viruses based on metagenomes and highlight the potential roles of diverse and unique gut viruses for the health of filter-feeding bivalves.

**IMPORTANCE:**

This study uses metagenome sequencing to establish the first scallop virome database. The study reveals previously unknown diversity of scallop-associated viruses and provides insights into links between disease status and viral diversity and genome content. The study will interest many aquatic virologists and could have important implications in managing marine resources.

## INTRODUCTION

Bivalves (within the mollusca phylum) are the most abundant and diverse group of marine animal species and play a crucial role in the operation of most marine ecosystems. Many bivalves are significant fishery and aquaculture species and models for researching ocean acidification, biomineralization, and adaptation to coastal environments amidst climate change (1). Scallops are filter-feeding aquatic organisms that can bioaccumulate pathogens from contaminated water, thereby presenting a potential infection hazard to human consumers. During filter feeding, scallops sequester viruses and accumulate them in their digestive system, especially in the intestines. Thus, viruses stored in the intestines of scallops can be used as indicators of environmental pollution and disease monitoring.

Viruses play multifaceted roles in marine ecosystems, driving host evolution through horizontal gene transfer (HGT), modulating symbiotic relationships via metabolic interference, and serving as key regulators of marine biogeochemical cycles (2–4). Global Ocean Virome 2.0 project (5) and advances in metagenomic sequencing have significantly expanded our understanding of viral diversity, particularly bacteriophages critical to microbial population dynamics (6–8). Among these advancements, the discovery of marine viruses, particularly those that infect bacteria, stands out as a notable contribution to our knowledge of marine ecosystems (9). Despite progress in marine virology, viral communities in economically vital bivalves like scallops (Pectinidae) remain understudied. While scallop microbiomes are known to be shaped primarily by endogenous factors (10–13), their viral counterparts—including both pathogenic and commensal viruses—lack systematic characterization. Outbreaks of scallop-specific pathogens (e.g., acute viral necrosis virus in *Chlamys farreri*) (14) highlight the urgent need to decipher scallop-virus interactions. Furthermore, anthropogenic pressures (e.g., coastal eutrophication) may introduce human-associated viruses (norovirus, hepatitis A virus) into scallop habitats, compounding ecological and health risks. It is anticipated that the study of scallop viruses will yield valuable insights into their ecological roles and health impacts. Additionally, determining whether scallop viruses play a critical role in disease prevention and control in cultured scallops, as well as in the assessment of marine microecological health, holds significant practical and economic importance for aquaculture.

In the summer of 2022, a mass mortality of scallops occurred at Sanggou Bay Scallop Breeding Center in Rongcheng, Weihai, China. In this study, 13 gut viruses of scallops of varying health status were examined. To further examine the role of viruses in the gut, a reference-independent method was employed to extract and assemble viral sequences, reducing interference from host and bacterial DNA. This methodology facilitated the precise delineation of pivotal viral clusters (VCs) among the scallop gut virome, enabling an in-depth investigation into their phylogenetic relationships and ecological dynamics.

## MATERIALS AND METHODS

### Scallop sampling

This study used adult *Chlamys farreri* scallops (25 + 5 mm in shell length), collected in May 2022 from Rongcheng, Weihai. The mortality of scallops was determined by randomly selecting 20,000 individuals from multiple suspended cages at random sites. Moribund scallops showed mantle atrophy and abscess formation on the adductor muscle, causing impaired contractility and eventual decay. Based on their phenotype, wild-type scallops had a survival rate of nearly 30%, while the Zhikong scallop variety “Penglaihong No. 3” cultured at the same location had a 100% survival rate. Thus, healthy “Penglaihong No. 3” scallops were referred to as the control group “SGW1L,” moribund wild-type scallops were referred to as the “SGW2D,” with the surviving wild-type scallops referred to as the “SGW2L” group. From these groups, four sequencing libraries were constructed from SGW1L, four sequencing libraries were constructed from SGW2D, and five sequencing libraries were constructed from SGW2L.

### Viral nucleic acid extraction and sequencing

The sequencing materials were derived from intestinal tissue samples in the “Scallop sampling” module. The total genomic DNA of the samples was extracted with the QIAamp PowerFecal Pro DNA Kit (Qiagen, Germany) following the manufacturer’s instructions, and the DNA concentration was determined using a Qubit fluorometer (Thermo Scientific, USA). Metagenome sequencing libraries were generated using the extracted DNA described above, and the high-throughput sequencing was accomplished on the Illumina PE150 platform (Novogene, Tianjin, China) with a depth of 40G to ensure that low-abundance viruses in the sample could be detected. DNA quality was assessed via agarose gel electrophoresis (for purity and integrity), Nanodrop (OD 260/280 ratios for purity), and Qubit 2.0 fluorometry (for accurate concentration). Qualified DNA samples underwent random fragmentation using the Covaris ultrasonic disruptor, followed by library preparation involving end repair, A-tailing, adapter ligation, and purification.

### Virus detection and quantification based on *de novo* assembly (viral operational taxonomic unit [vOTU] annotation)

We used Bowtie2 (15) to align the sequencing results to the scallop genome, excluded host DNA contamination, and retained unmatched pairs of reads for subsequent viral metagenomic analysis. High-quality reads were trimmed using fastp v.0.20.0 (16) with options for correction, polyG and polyX trimming, overrepresentation analysis, and adapter detection. MEGAHIT v.1.2.9 (17) was used for merging and assembling reads into contigs over 1,500 bp. Contigs ≥1,500 bp from metagenome assemblies were used to recover viral sequences by geNomad (18) and VirSorter2 v.2.2.4 (19) using default settings, following specific categorization and scoring criteria. In total, 95,169 viral contigs were identified. Viral contigs were clustered into 38,288 vOTUs at 95% nucleotide identity across coverage of target -c 0.8 using mmseqs2 (20), of which 1,564 were ≥10 kb.

### Generation of prokaryotic macrogenome assembly genomes

The contigs from the MEGAHIT assembly were processed through metaWRAP (21) for binning, refinement, and reassembly. Binning involved metabat2, maxbin2, and concoct, selecting bins with ≥50% completeness and ≤10% contamination. These bins were refined and reassembled at a finer scale. To reduce redundancy, dRep (22) was employed (parameters: -comp 50, -con 10, -sa 0.95), resulting in metagenome-assembled genomes (MAGs) representing the prokaryotic hosts. MAGs were taxonomically assigned using GTDB-tk v.2.3.2 (23) based on classify_wf workflows. Maximum-likelihood phylogeny of MAGs was inferred using IQ-TREE v.2.2.0.3 (24) from a concatenation of 120 bacterial or 122 archaeal marker genes produced by GTDB-tk v.2.3.2 (25); the generated tree was visualized using iTOL v.1.8 (https://itol.embl.de/). To determine the relative abundance of MAGs in each sample, clean reads were mapped to MAGs using CoverM v.0.6.1 (26) (with parameters -contig -m rpkm --trim-min 5 - -trim-max 95) to calculate reads per kilobase of exon model per million mapped reads (RPKM) values.

### Viral genome integrity, taxonomy, and auxiliary metabolic gene (AMG) analysis

Viral genome completeness in contigs was assessed using CheckV v.1.0.1 (7, 27). Contigs primarily aligning with intestinal microbial genes rather than viral genes were identified as false positives and removed, yielding 268 viral contigs with over 90% genomic completeness. Family-level viral contig classification was performed using VITAP (28), an unpublished method compatible with the Baltimore Classification Framework. CheckV also determined viral boundaries and removed host sequence contamination prior to annotation. Annotation primarily used DRAM-v (29) models, requiring VirSorter2 output. All vOTUs were prepared for annotation using VirSorter2 (--prep-for-dramv). The AMGs in vOTU sequences were identified by VIBRANT using default parameters. DRAM-v annotations, supplemented by manual curation, identified AMGs and confirmed their features using NCBI CD-Search (30) and Phyre2 for structural homology. Genomic maps for AMG-encoding contigs were created using DRAM-v and VirSorter2 annotations. A gene was regarded as an AMG candidate if assigned to a metabolic module and/or to a previously described AMG and had an auxiliary score (confidence of virus-encoded)  ≤3. This led to the establishment of a permissive AMG catalog. Firstly, an AMG was only kept if it was located within virus regions called by CheckV. Secondly, we screened for non-virus regions by checking for sequences adjacent to phage genome ends, including tRNA regions and inverted/direct repeats. tRNA regions were detected by tRNAscan-SE (31) using general tRNA models. Inverted and direct repeats were predicted using the application inverted in EMBOSS (32) with standard qualifiers and -scircular1 for circular virus contigs. Thirdly, we removed AMGs on virus contigs containing mobile genetic elements and other genes that may facilitate the random integration of microbial metabolic genes. AMGs were excluded if they were found on contigs carrying genes encoding transposons, lipopolysaccharide islands (glycosyltransferase, nucleotidyl transferase, carbohydrate kinases, and nucleotide sugar epimerase), endonucleases, integrases, or plasmid stability genes.

### Prediction of antimicrobial resistance genes, virulence factors, and toxins

All vOTUs used Diamond BLASTp v.2.1.8.162 (33) for protein sequence comparison against the virulence factors of bacterial pathogens (VFDB) database (https://www.mgc.ac.cn/VFs/main.htm) (parameters: -evalue 1e-10 -max-target-seqs 1) and hmmsearch 3.4 (34) to search for genes related to pathogens that may be contained in the viral sequences to extract the true virulence factor sequences. Concurrently, we employ the PathoFact 2.0 integrated database to predict antibiotic resistance genes, virulence factors, and toxins within the data set, subsequently consolidating the acquired results. The toxin-antitoxin (TA) system proteins were retrieved from TADB 3.0 (https://bioinfo-mml.sjtu.edu.cn/TADB3/), and a database was constructed using the BLASTp module of the Diamond software. Viral proteins were then compared against this database to identify toxin and antitoxin genes within the TA system (35).

### Calculation of relative virus abundance and host prediction

Virus and host relative abundances were quantified using CoverM (26), with criteria of minimum 95% read identity and 75% aligned read percentage. For clarity, RPKM values were normalized to transcripts per kilobase of exon model per million mapped reads. Post-MAGs acquisition, viral hosts were predicted based on clustered regularly interspaced short palindromic repeats (CRISPR) spacer sequences. The software ARAGORN (36) was utilized to identify tRNA sequences within vOTUs and MAG sequences. All identified tRNA sequences were subjected to bidirectional comparisons using blastn. Only when the bidirectional comparison results of tRNA sequences from vOTUs and MAGs demonstrated more than 90% consistency and coverage were they considered indicative of a potential virus-host relationship. To investigate HGT between viruses and bacteria, and between viruses and bivalves, we analyzed high-quality viral genomes (scallop virome data set [SVD]) selected based on strict completeness and contamination criteria. Viral proteins from the SVD genomes were systematically compared against this cellular nr database using Diamond BLASTp with stringent parameters: E-value cutoff 1 × 10^−50^, minimum 50% alignment coverage, and 50% sequence identity. To eliminate lineage-specific homology, best hits sharing taxonomic lineage with query sequences were excluded. Potential HGT candidates were identified when cellular database hits showed significantly stronger alignment confidence (lower E-values) compared to matches within the nr virus protein database. For network construction, bit-score values from Diamond BLASTp alignments were used to define edge weights. After conducting functional annotation of HGT candidates through integrating Pfam domain predictions and eggNOG Mapper (v.1.0.3) categorization, we utilized Cytoscape (v.3.9.1) to carry out the visualization of HGT networks.

## RESULTS

### Overview of the SVD

**Fig 1:**
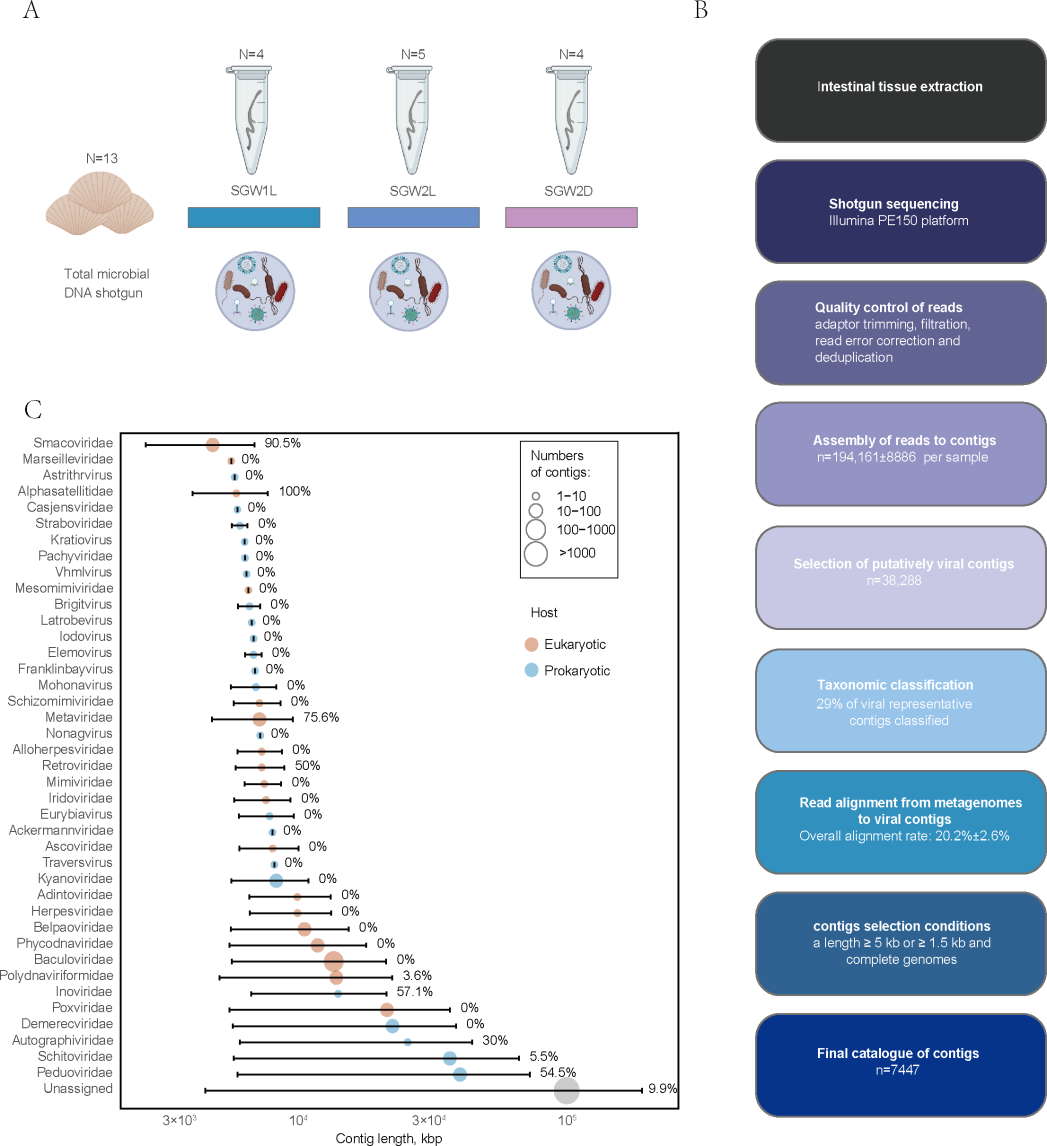
Experimental design and distribution of viral representative contigs by length and taxonomic family. (A) Intestinal sample collection from 13 study subjects and types of analyses performed. Each color block represents a different sample type. (B) Overview of the experimental protocols and bioinformatic pipelines. (C) Distribution of 7,447 viral representative contigs by length, taxonomic family. Segments represent the spread between the minimal and maximal lengths of contigs assigned to the taxonomic family rank. Dot size represents the number of contigs within the taxonomic family rank. Numbers opposite each segment represent the percentage of circular contigs among the contigs within the taxonomic family rank.

To determine the diversity and variations in the gut viruses of scallops in different states of health, a metagenomic analysis of the gut viruses of 13 Zhikong scallops (*Chlamys farreri*) was conducted. Scallop intestinal tissue samples from the aquaculture regions of Sanggou Bay, a typical Chinese scallop aquaculture area, were analyzed (Fig. S1A). The healthy samples from two different kinds of scallop germplasms collected from Sanggou Bay were named SGB1L and SGB2L, respectively; the moribund sample of germplasm 2 was named SGB2D (Fig. 1A). Sparse curve analysis confirmed high sequencing coverage (Fig. S1B). A total of 2,500,483 contigs (≥1,500 bp) were assembled, of which 95,169 viral contigs were identified. After redundant contigs were removed through alignment, 38,288 vOTUs were classified. Only 0.7% (*n* = 268) of the 38,288 vOTUs were classified as complete or high-quality viral genomes with over 90% genomic completeness by CheckV (Fig. S1C). For the following analysis, the 7,447 vOTUs that were ≥5 kb or ≥1.5 kb with complete genomes were retained and used (Table S1). Viral composition of metagenomes was established using a *de novo* assembly-based approach (Fig. 1B; Fig. S2). To gain a more complete view of the composition of the viruses, the VITAP pipeline was used. This approach allowed taxonomic assignment to four orders approved by the International Committee on Taxonomy of Viruses (ICTV) for 27.7% of our 7,447 vOTUs. Among the classifiable viruses, double-stranded DNA (dsDNA) eukaryotic viruses accounted for 74.7%, single-stranded DNA (ssDNA) eukaryotic viruses accounted for 3.8%, dsDNA prokaryotic viruses accounted for 20.4%, and ssDNA prokaryotic viruses accounted for 1.2%.

### Stability and novelty of scallop gut viruses

**Fig 2:**
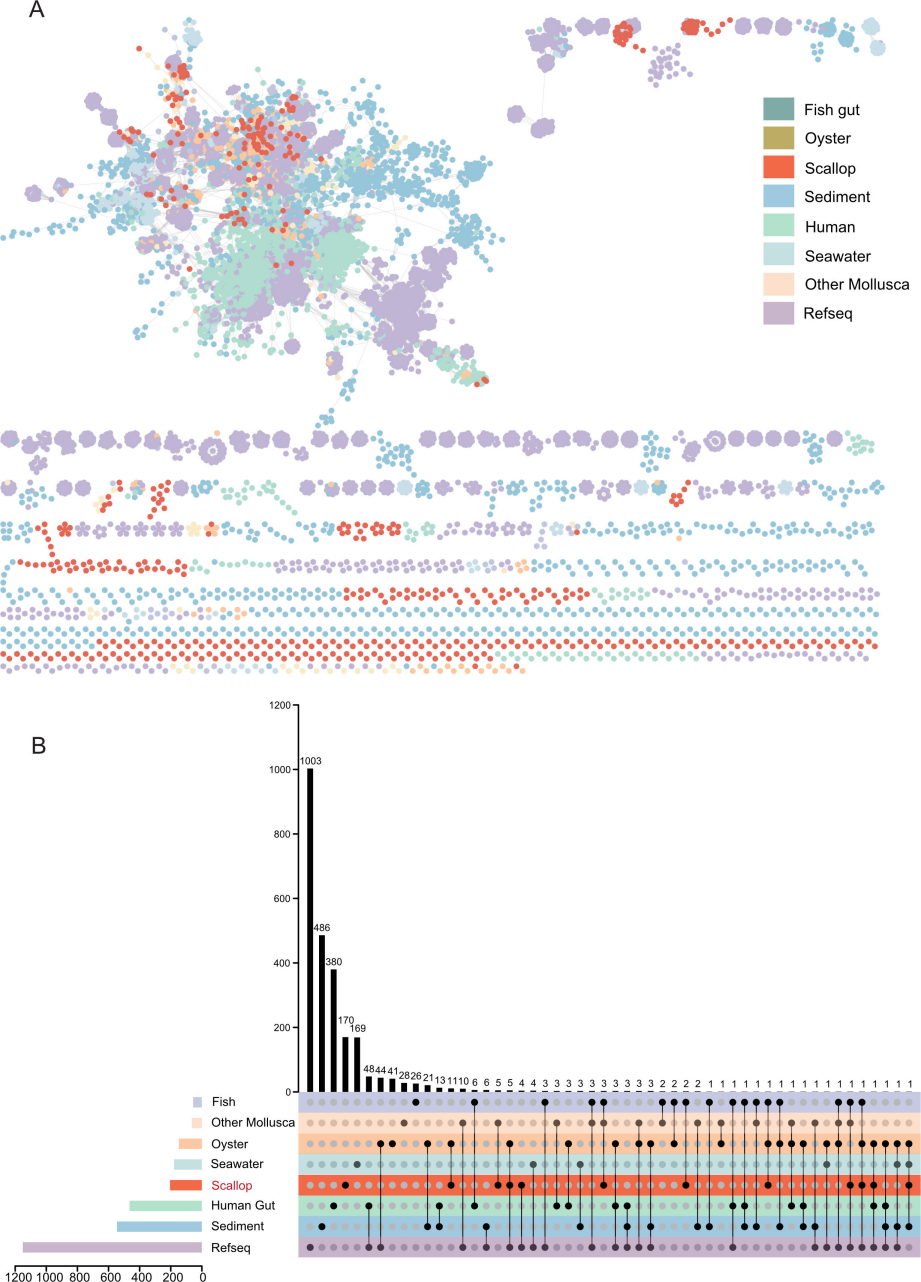
Comparative analysis of scallop enteroviruses with other viruses found in marine habitats and other enteric environments. (A) Gene-sharing network of viral sequence space based on assembled viral genomes from scallop, oyster, seawater (from IMG/VR v4), fish gut (IMGVR), sediment (IMGVR), human gut, other mollusks (IMGVR), and NCBI RefSeq viral genomes. (B) UpSet plots show virus clusters shared between the scallop gut data set and other data sets. Source data are provided as Table S4.

At the family level, the number of classified vOTUs of SVD predominantly comprised *Revtraviricetes* (*Belpaoviridae n* = 70, *Metaviridae n* = 78, and *Retroviridae n* = 6) and viruses infecting arthropods, such as *Polydnaviriformidae* (*n* = 83) and *Baculoviridae* (*n* = 103) (Fig. 1C). We mapped the abundance of reads using viruses from the Shellfish Major Pathogens Database (https://www.southchinafish.ac.cn/yubing.htm) to compare the differences in pathogenic viruses between the moribund and healthy groups (Fig. S3). *Herviviricetes* and *Revtraviricetes* viruses were found mainly in the scallop gut, and unlike the homogeneously distributed *Herviviricetes*, *Revtraviricetes* were only enriched in the moribund group. This differs from oysters (37), indicating that distinct viral assemblages are present in different mollusks. vConTACT2 (25) networks constructed with vOTUs and NCBI RefSeq viral genomes longer than 10 kb showed that they have the same topology (Fig. S4A). The SVD contributed 23.86% to the vConTACT2 network’s nodes, highlighting gaps in the understanding of the ocean virosphere (Fig. S4A). To further investigate the diversity of viral communities in scallops and their relationships with viral sequences identified from other relevant environments, a gene-sharing network was constructed using vConTACT2. This weighted network can classify viral sequences into VCs corresponding to genus-level groups. The vOTUs identified from scallops, oysters (37), human gut (38), fish gut (Integrated Microbial Genome/Virus Resource ([IMGVR]), sediment (IMGVR), environmental seawater (IMGVR), other mollusks (IMGVR), and RefSeq viruses were clustered into 2,476 VCs (Fig. 2A). Additionally, oysters, fish gut, human gut, seawater, sediment, other molluscan viruses, and NCBI RefSeq viruses contributed 148, 56, 465, 179, 546, 65, and 1,151 VCs, respectively (Fig. 2A). In the case of scallop intestines, 537 vOTUs were clustered into 205 VCs, with the majority (82.3%) having no homologs in existing reference databases. This suggests that most scallop intestinal viruses are unique to this habitat. Among the 205 scallop intestinal VCs, only 11 VCs were shared with oysters, 8 VCs with fish gut, 1 VC with sediment, 1 VC with the human gut, 9 VCs with other mollusks, and 12 VCs with RefSeq viruses. The remaining 170 VCs of viruses were exclusive to scallop viruses, potentially representing candidate novel genera (Fig. 2B). These scallop-specific VCs contained 402 vOTUs, with the most abundant being *Caudoviricetes* (*n* = 63, 15.7%), *Naldaviricetes* (*n* = 4), and *Revtraviricetes* (*n* = 2). The remaining 333 vOTUs could not be taxonomically assigned at the family or higher level at this time.

### Novel viral contigs and potential animal infections

The length of 268 complete or high-quality viral genomes ranged from 2,370 to 189,007 bp and had GC contents between 28.86 and 52.97% (Table S2). The majority of these vOTUs with assigned taxonomy belonged to *Metaviridae* (22%) and *Smacoviridae* (7%) (Fig. S5A). Remarkably, almost all smacoviruses identified in the SVD were of high quality. In SVD, 7,447 vOTU-encoded proteins showed 0%–99.3% identity with known viral proteins, primarily in the 25%–50% range, indicating many represent new viral categories (Fig. S5B). Seventy-one percent (5,256) of the 7,447 vOTUs were unknown or unclassified sequences and classified as novel viruses at the class level. The classified genomes spanned 13 DNA virus classes and 40 families, with the notable presence of *Caudoviricetes*, including families like *Schitoviridae* (36), *Demerecviridae* (25), *Kyanoviridae* (15), *Peduoviridae* (11), *Autographiviridae* (10), and others.

Of the identified viral families, *Smacoviridae*, *Adintoviridae*, *Baculoviridae*, and *Orthoherpesviridae* showed potential to infect animals, including humans. *Smacoviruses*, not yet cultured and with unconfirmed hosts, may infect gut-associated methanogenic archaea (39). *Adintoviridae* infects a range of vertebrates, from fish to humans, across six genera (40). *Baculoviridae* primarily targets insects such as Lepidoptera, Hymenoptera, and Diptera (41). *Orthoherpesviridae* infect humans and other vertebrates. The presence of potential avian viruses suggests that scallops could be important reservoirs and transmission vectors for these marine viruses, particularly considering the risk of water contamination via bird feces.

### Unique diversity and phylogeny of *Smacoviridae* in scallop gut viruses

*Smacoviridae*, identified in the fecal metagenomes of insects and vertebrates, are part of the CRESS-DNA virus family (42). These viruses have genomes approximately 2.5 kb in size and primarily encode for replication initiator protein (Rep) and capsid protein genes. While they are proposed to infect eukaryotes, their hosts remain undetermined. *Smacoviridae*-related branches are intermixed with unannotated ones, suggesting uncertainty in their classification (Fig. S5A). Additionally, smacoviruses show distant relationships to other CRESS-DNA viruses, known to infect both plants and animals, highlighting their unique placement within this virus family.

**Fig 3:**
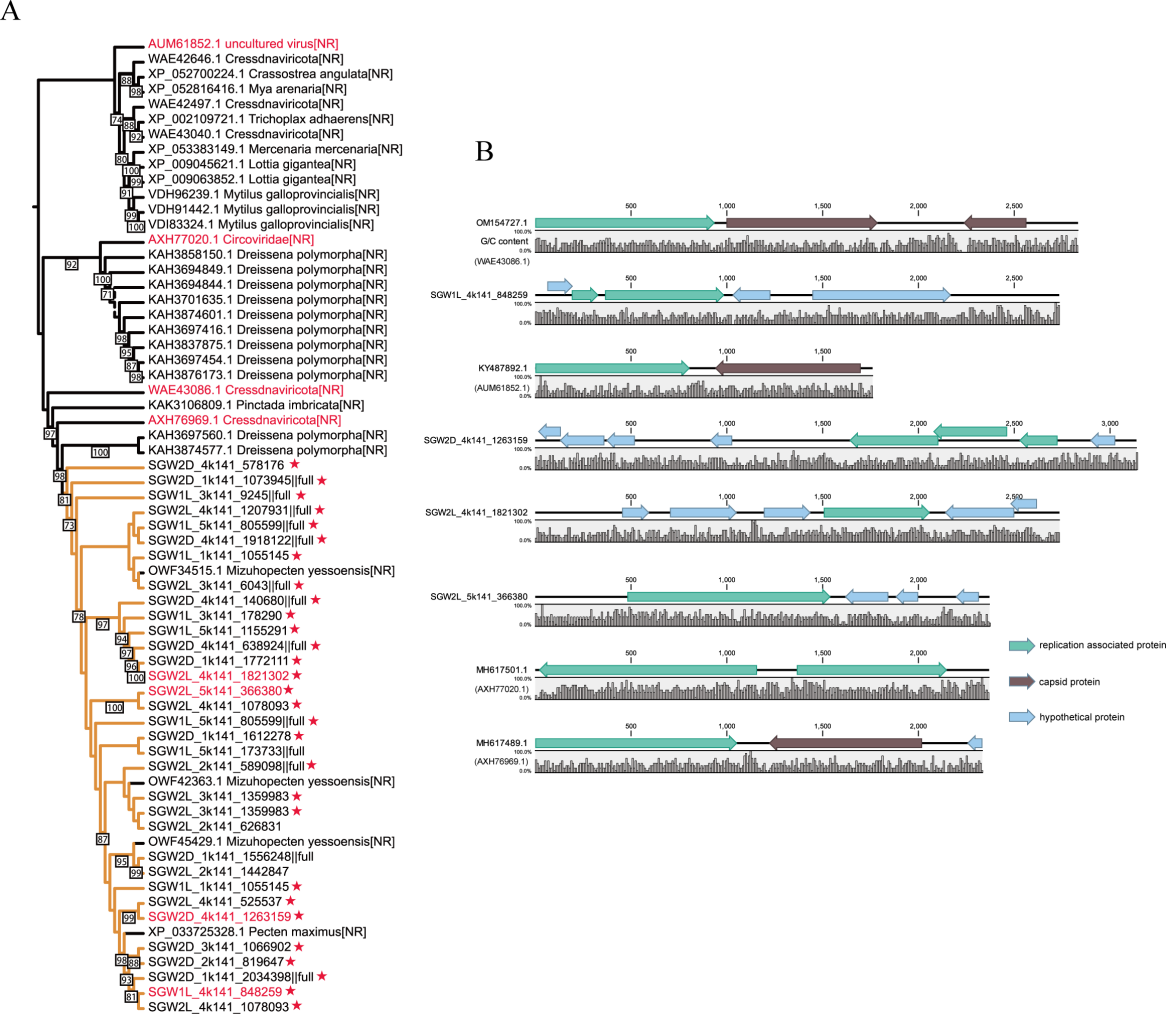
Phylogeny of replication initiator proteins of scallop-related *Smacoviridae*. (A) The large tree on the left shows the phylogeny of selected proteins in the NR of the full rep and of the SVD, with bootstrap values displayed on the branches, and only values above 70 are shown. Branch colors represent data sources: orange, SVD; black, NCBI nr database; red stars, SVD high-quality sequences; red tree node ID sequences are used to show gene mapping. (B) Demonstration of genomic context. Tag colors indicate different gene annotation types: green, replication-associated protein; blue, hypothetical protein; brown, capsid protein.

Using *rep* sequences from smacoviruses cataloged by the ICTV, 1,836 and 33 *rep* sequences were identified in the NCBI nr database and the SVD data set, respectively, suggesting the high diversity of smacoviruses present (Fig. S6). Phylogenetic analysis revealed that SVD *rep* sequences form a unique branch. In the same phylogenetic branch, shellfish sequences labeled as *Dreissena polymorpha* and *Mizuhopecten yessoensis* were identified. Together with a representative from SVD, these sequences form a distinct branch separate from certain *Cressdnaviricota*, such as *Circoviridae*, suggesting a shared genetic origin (Fig. 3A). Tblastn searches reveal that SVD *rep* sequences are homologous to the replication-associated protein of *Cressdnaviricota*, indicating their viral origin. Another possibility is that horizontal gene transfer between *Smacoviridae* and other mollusks occurred. To understand the structure of these sequences, the complete sequences of this branch were selected for presentation (Fig. 3B). The gene map shows that all sequences contain the replication-associated protein, although their positions in the genome vary. Some spliced sequences from the SVD have lost the capsid protein and instead acquired a hypothetical protein. Further investigation is needed to understand the pathogenicity of these smacoviruses, their interactions with scallop hosts, and the potential for cross-species transmission.

### Diversity analysis of virus community structure

**Fig 4:**
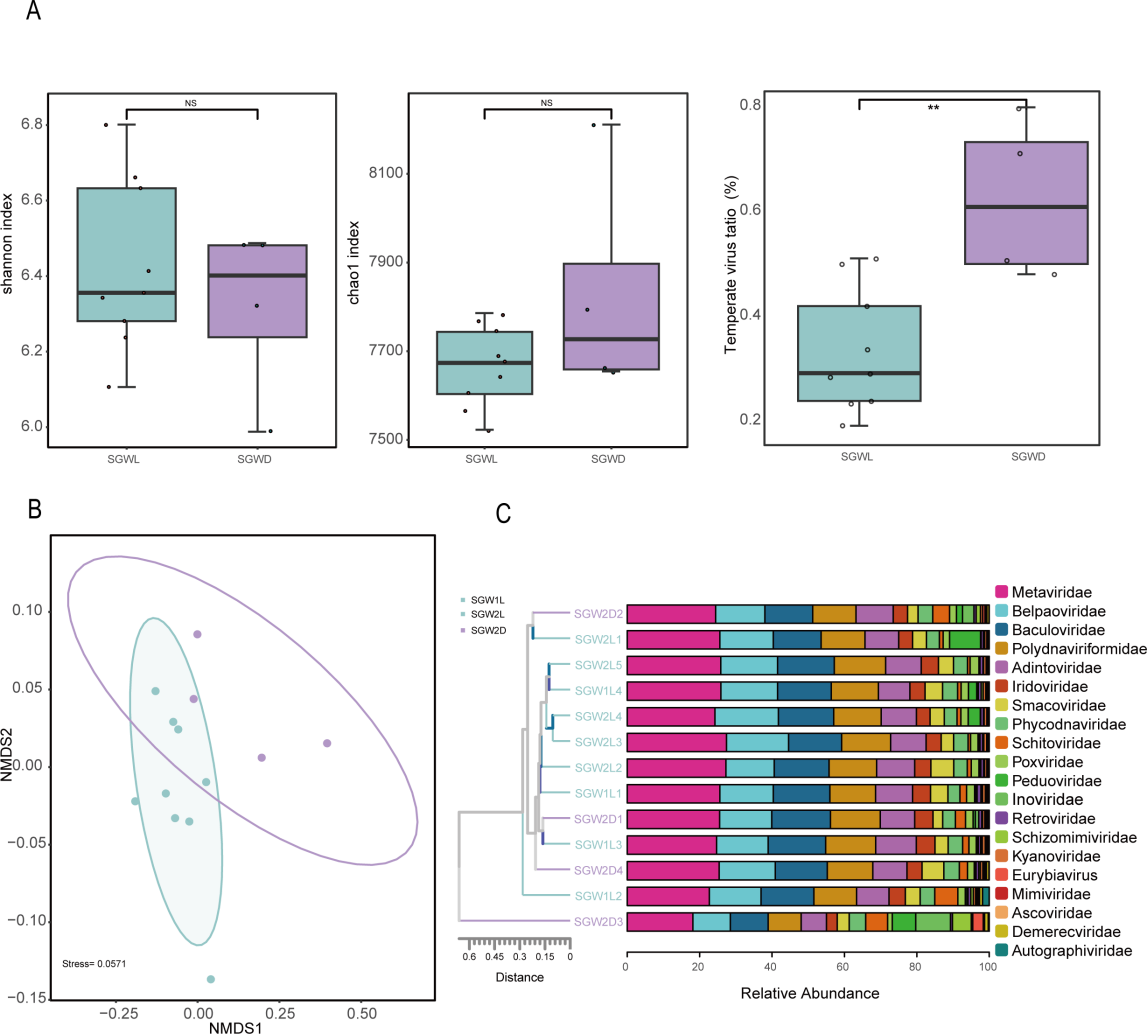
Viral community in the SVD. (A) Shannon, Chao1 indices of the viral community diversity from healthy and moribund samples, temperate virus ratio of count of different lifestyle viruses in different groups. (B) NMDS analysis of a Bray-Curtis dissimilarity matrix calculated from RPKM values of vOTUs. Analysis of similarities (ANOSIM) was applied to test the difference in viral communities about health status. SGB-L, healthy samples; SGB-D, moribund samples. (C) Relative abundance of viruses in each scallop intestine sample at the family level obtained from metagenome data set.

Viral communities in scallops were further analyzed to both determine whether they formed a cohesive group or represented a random assembly, and also to evaluate their response to environmental changes. The diversity indices showed no significant difference between healthy and moribund scallop populations (Fig. 4A). The viral lifestyle was determined based on the results of CheckV and manual checking for the presence of lysogeny-specific genes. The percentages of temperate viruses were significantly higher in the moribund group than in the healthy group (Fig. 4A). These bacteriophages not only increased the instability of the bacterial community through the lysis cycle, but also enhanced the pathogenicity of pathogenic bacteria through the transmission of virulence factors and AMGs. Thus, changes in the abundance of temperate viruses in the moribund group are both the result of virus-bacterial interactions and may be a driving force for disease exacerbation. Non-metric multidimensional scaling (NMDS) analysis shows significant differences in viral community structure between these groups (Fig. 4B), suggesting subtle differences in their viral community composition. Here, it is shown that of the top 20 most abundant viruses at the family level, *Revtraviricetes* was still the most abundant (Fig. 4C). Combined with the subsequent comparison of shellfish pathogen data, it was speculated that *Revtraviricetes* may be one of the main viruses in moribund scallops. See Fig. S3 for details. In *Crassostrea gigas*, OsHV-1 μVar virus predominates during outbreaks, reducing viral diversity, but this study did not find such disparities between moribund and healthy scallops, indicating that OsHV-1 μVar may not be the primary pathogen in the scallop samples.

### Virus-host linkages

**Fig 5:**
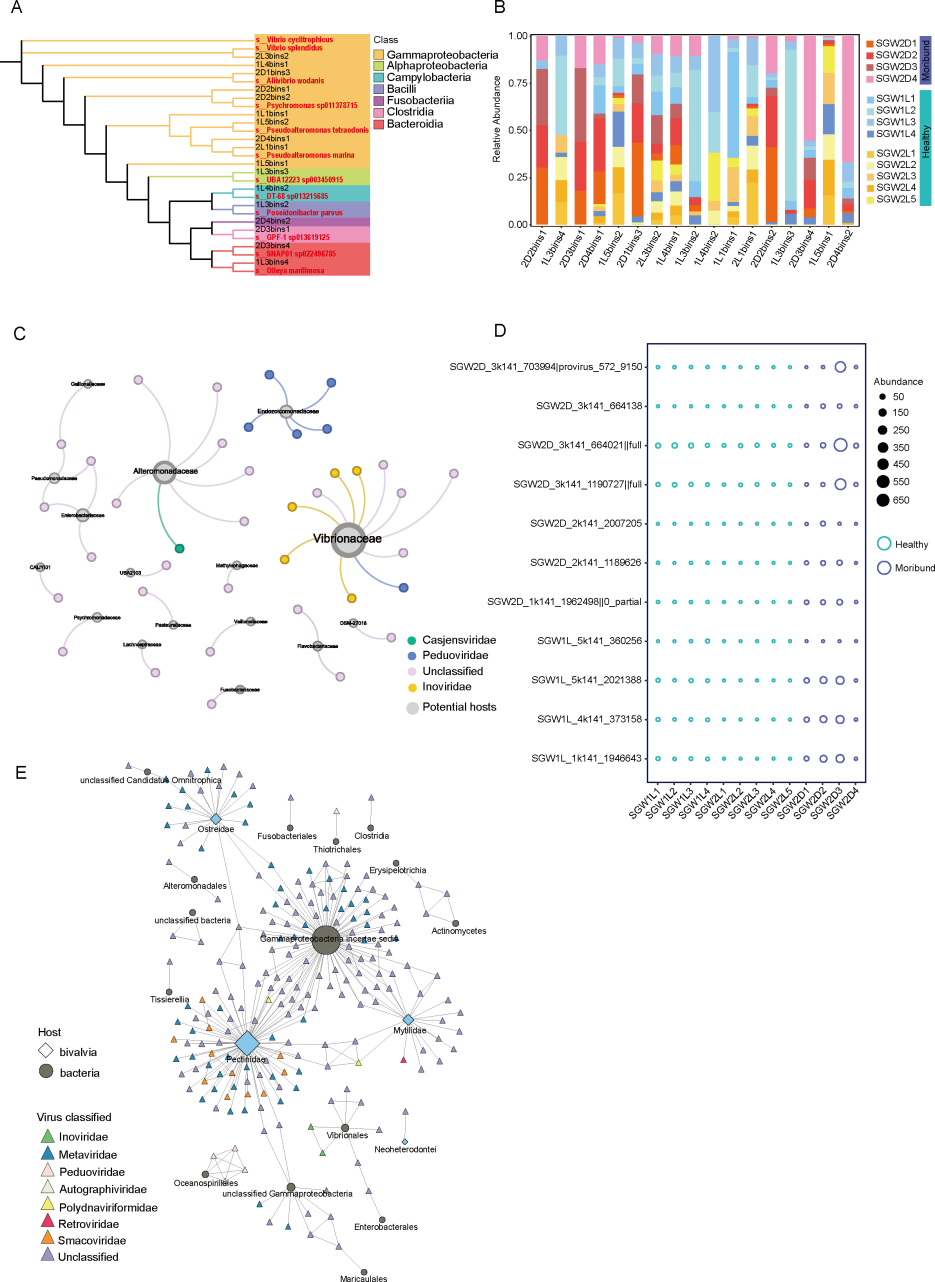
Predicted host-virus interactions. (A) Maximum-likelihood phylogenetic tree of prokaryotic MAGs. The phylogenetic tree was inferred from the concatenated alignment of 120 bacterial or 122 archaeal single-copy marker genes. Clades are colored according to their annotated class, and the names of reference genomes and scallop gut MAGs are presented in red and black text color, respectively. (B) Relative abundance statistics of MAGs at different stations. (C) Predicted virus-host linkages. The gray circles represent potential hosts, the hosts and viruses are classified by family, the lines represent the number of viruses predicted for that host, and the virus families are represented by different colors. (D) Pattern of abundance distribution of vOTUs infecting potential *Vibrio* families. Source data are provided as a source data file. (E) Undirected HGT networks of viruses and prokaryotes and bivalves with nodes representing previously described virus families and names labeled below the nodes; edges are weighted according to the number of detected transfers.

*De novo* assembly and binning of metagenomes resulted in 34 high- or medium-quality microbial MAGs with completeness ≥50% and contamination ≤10%. These MAGs were then clustered at 95% average nucleotide identity to generate 17 bacterial MAGs, representing species-level groups spanning six phyla (Fig. 5A). In these samples, bacterial MAGs were dominated by Gammaproteobacteria (10). Others included Alphaproteobacteria (1), Bacteroidia (2), Campylobacteria (1), Bacilli (1), Fusobacteriia (1), and Clostridia (1). Based on the read coverage of MAGs among samples, it was found that while most of the MAGs were present in all 13 scallop samples, some of the MAGs were specific to certain samples; for example, 2D3bins1 (Tissierellales) was only present at site SGB2D, and 1L4bins2 (*Metamycoplasmataceae*) was only present at the SGB1L and SGB2L sites, respectively (Fig. 5B). Consistent with prior studies (43), the comparison of CRISPR spacer sequences indicates that the majority of vOTUs are likely to have a narrow host range. Only 41 vOTUs are linked to bacterial hosts, with most linking to *Vibrio* family hosts. *Vibrio* infection is intricately associated with fatal diseases in scallops (44). Additionally, three vOTUs are predicted to have the potential for cross-family infection, but they have not yet been identified at the family level (Fig. 5C). Linear discriminant analysis effect size analysis identified specific biomarkers in different samples: P_Campylobacterota in SGB1L, P_Fusobacteriota in SGB2D, and O_Gammaproteobacteria in SGB2L (Fig. S7). Since *Vibrio* is predicted to be the most common putative host and also one of the key pathogenic factors of shellfish, the relative abundance of 11 vOTUs linked to *Vibrio* in each sample was calculated (Fig. 5C). These viruses were more abundant in the moribund group of scallops, as the moribund scallops contained more abundant *Vibrio*. Correspondingly, the virus abundance changed accordingly (Fig. 5D). These results suggest varying microbiome responses related to the health conditions of scallops.

To further investigate potential interactions between viruses and prokaryotes, as well as between viruses and bivalves (class Bivalvia), we conducted HGT analysis using high-quality viral genomes selected from the scallop gut virome. Our study revealed linkage information for 237 putative HGT-derived genes across 7 viral families, 15 prokaryotic groups, and 4 eukaryotic lineages (Fig. 5E). Among classified viruses, we identified 30 HGT linkages connecting viral genomes to prokaryotic microorganisms, including *Peduoviridae* infecting Gammaproteobacteria (specifically Oceanospirillales) and *Inoviridae* associated with *Vibrionales*. The family *Metaviridae* exhibited the broadest host range among analyzed viruses. These retrotransposon-related viruses were predominantly detected in prokaryotes and eukaryotes, with notable prevalence in plants and invertebrates.

Notably, unclassified viruses demonstrated dual linkages to both prokaryotes and bivalves, carrying genomic fragments showing high sequence homology to bacterial genomes while simultaneously sharing similarities with bivalve and other eukaryotic genomes. This observation suggests their potential role as mediators of cross-kingdom genetic exchange. In virus-bivalve interactions, *Smacoviridae* showed concentrated associations with the *Pectinidae* lineage, indicating specialized host-virus dynamics consistent with our previous phylogenetic analyses of *Smacoviridae*-scallop relationships. Additionally, minor associations were observed between viral clusters and *Ostreidae* or *Mytilidae* lineages.

This comprehensive analysis delineates complex interaction networks spanning multiple bacterial phyla and bivalve species across diverse viral families. The identified cross-phylum and cross-kingdom transmission patterns underscore the evolutionary significance of broad host-range adaptability in viral evolution and ecological niche colonization.

### Viral AMGs in healthy and moribund groups

**Fig 6:**
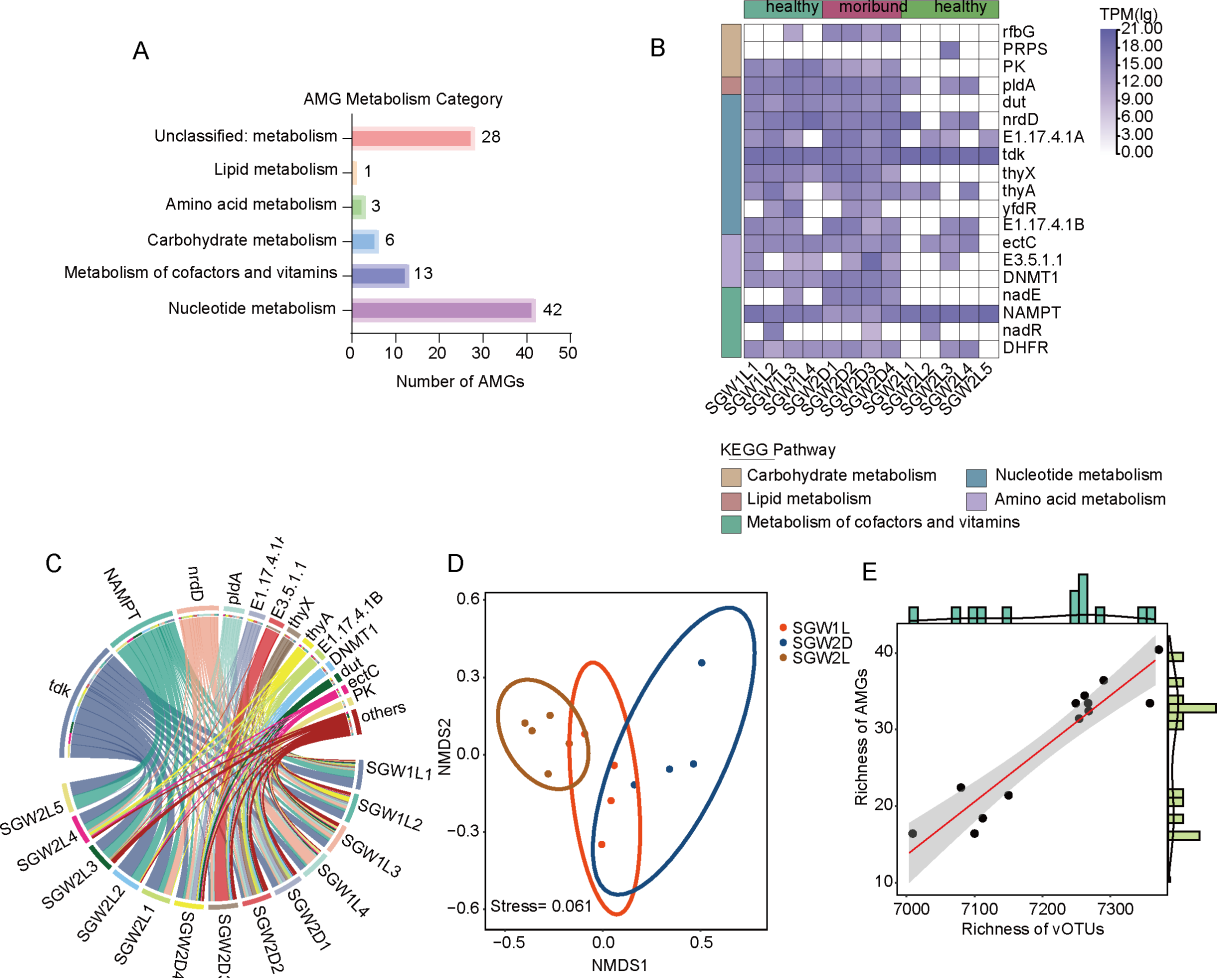
Virus-carried host metabolic genes correlate with scallop health status. (A) Quantitative display of AMGs of different metabolic pathways. (B) Relative abundance of viral AMGs obtained from metagenome data set in different scallop intestine samples. KEGG metabolic categories are colored according to the legend. (C) Percentage of AMG abundance in each sample. (D) NMDS plot of AMG diversity in the SVD libraries (*n* = 13). (E) Correlations and linear correlation curves of the richness indexes between vOTUs and AMGs.

Viruses play important roles in regulating metabolic processes within the marine ecosystems (45). To better understand the impacts of viral AMGs on host metabolisms and relevant biogeochemical cycling, AMGs were identified from vOTUs by VIBRANT and DRAM-v pipelines; they were further functionally annotated with Pfam and Kyoto Encyclopedia of Genes and Genomes (KEGG) databases. Here, 93 AMGs within the SVD data set have been identified and classified across nine different metabolic pathways (Table S3; Fig. 6A). They show significant enrichment in the metabolic pathways related to cofactors and vitamins, amino acids, lipids, nucleotides, and carbohydrates. (Fig. 6B and C). The moribund group showed enrichment in amino acid metabolism, while the healthy group focused instead on carbohydrate metabolism. The abundance of AMGs associated with amino acids and carbohydrate metabolism underscores their potential influence on host metabolic processes. This trend aligns with observations from other marine viruses (46, 47), underscoring the critical role of viruses in marine ecosystem metabolism. Additionally, there was a strong congruence and positive correlation in richness and diversity between the AMG and vOTU communities (Fig. 6D and E). This suggests a significant relationship between functional and species diversity within the scallop viruses, enhancing our understanding of its ecological role in scallops.

### Scallop gut harbors abundant antimicrobial resistance genes, virulence factors, and toxins

In the refined exploration of the SVD, 50 virulence factors traditionally associated with bacterial pathogens were identified (Fig. S8A). These factors were found within the scallop viruses, suggesting the potential role of bacteriophages in mediating the horizontal gene transfer of pathogenic determinants. This phenomenon underscores the capacity of bacteriophages to act as vectors for virulence factors, thereby enhancing the pathogenic potentials of their bacterial hosts within scallop populations. Notably, UDP-N-acetylglucosamine pyrophosphorylase from *Francisella noatunensis*, relevant to aquaculture and fish health, was enriched in the moribund group, causing diseases such as francisellosis in fish (48). In addition, there are a number of virulence factors derived from *Vibrio* that were enriched in the moribund scallops, such as *Vibrio vulnificus* and *Vibrio fischeri, Campylobacter jejuni*, *Bacillus cereus*, *Yersinia enterocolitica,* and *Yersinia enterocolitica*. These detected foodborne disease bacteria can cause gastroenteritis and food poisoning. These bacteriophage-carried virulence factors not only facilitate bacterial colonization and persistence but also modulate host immune responses and nutritional metabolism, contributing to disease manifestation and progression. This intricate interaction between bacteriophages, bacteria, and scallops highlights the significant impact of viral elements on the microbial ecology and pathogenic landscape within marine environments. We also identified 65 antibiotic resistance genes, among which those encoding vancomycin resistance regulatory proteins, chloramphenicol resistance proteins, and tetracycline resistance proteins showed significant enrichment, suggesting that viruses may serve as a reservoir for environmental resistance genes and reflecting the complexity of horizontal gene transfer in the intestinal environment of scallops. In the moribund group, some resistance genes related to efflux pump mechanisms were enriched, such as efrA and imrD, which might indicate that the host’s stress response is exerting selective pressure, leading to the selective enrichment of these resistance genes (Fig. S8B). Through interrogation of the TADB database, we identified 1,449 toxin-antitoxin (TA)-associated proteins exclusively derived from type II TA systems. As the most prevalent and phylogenetically diverse TA system, type II systems are characterized by bicistronic operons encoding proteinaceous toxin-antitoxin pairs that form non-toxic complexes through direct molecular interactions. Our analysis revealed four distinct toxin proteins and eight antitoxin counterparts, along with associated transcriptional regulatory elements and nucleic acid metabolism-related proteins involved in this pathway. Notably, empirical studies have demonstrated that type II TA systems exhibit rapid response mechanisms under cellular stress conditions, facilitating toxin activation through dissociation from antitoxin complexes, thereby enabling liberated toxins to engage their cellular targets and induce cytotoxic effects (Fig. S9). Our analysis underscores the critical need for a virus-centric approach to fully understand and address the molecular underpinnings of disease in aquaculture, paving the way for novel intervention strategies that target the viral vectors of bacterial virulence.

## DISCUSSION

Here, the SVD provides new insights into marine viruses via an in-depth study of DNA viruses in *Chlamys farreri*. This data set significantly expands our knowledge of viral diversity in mollusks, particularly semi-sessile bivalves. The analysis includes read recruitment, vOTUs, high-quality virus genomes, and smacovirus-related Rep proteins and uncovers a diverse and distinct viral ecosystem in scallops. Comparative analysis with oyster viruses reinforces this discovery. Scallop behavior and habitat suggest they are pivotal reservoirs and transmitters of marine viruses. The widespread presence of smacovirus Rep sequences in bivalve genomes underlines the prevalence of this viral group. Scallop viral communities also exhibit a structured nature, responding to host physiological changes and demonstrating a non-random compositional and functional organization.

While our study primarily focuses on scallop gut viruses, it is important to note that the broader context of microbial interactions is integral to understanding viral dynamics. We underscore the ecological importance of bivalves and their potential to accumulate pathogens from the environment. This context is crucial for framing the significance of studying viral communities in scallops, particularly in relation to disease outbreaks and environmental monitoring.

In scallops, health status was reflected by differential enrichment of AMGs. Moribund scallops showed an enrichment in amino acid metabolism and cofactor and vitamin genes, indicating metabolic disruptions due to viral infections. Healthy scallops exhibited fewer AMGs, focusing on secondary metabolite biosynthesis and carbohydrate metabolism, suggesting normal physiological states. The observed metabolic alterations in moribund scallops might be linked to energy imbalances and protein synthesis changes due to viral infections. The healthy scallops’ metabolic pathways imply regular energy metabolism and possibly enhanced environmental adaptability or defense mechanisms. Continued exploration of the structural and functional dynamics of bivalve viral communities is crucial to understand their impact on coastal microbiomes, disease dynamics, ecosystem health, and restoration efforts. Furthermore, by concentrating on markers that are strongly associated with known pathogens and virulence factors in diseased scallops, the development of specific probes in conjunction with conventional pathogen detection methods may facilitate comprehensive early diagnosis of health issues in scallops. This approach could lead to the establishment of a rapid detection system based on specific viruses or genetic markers.

## Data Availability

The raw sequence data reported in this paper have been deposited in the Genome Sequence Archive in the National Genomics Data Center, China National Center for Bioinformation/Beijing Institute of Genomics, Chinese Academy of Sciences (GSA: CRA020266) and are publicly accessible at https://ngdc.cncb.ac.cn/gsa. The analysis scripts used in this study are available at https://github.com/lijiaxiang111/Chlamys-farreri-scripts.

## SUPPLEMENTAL MATERIAL

Fig. S1 msystems.00342-25-s0001.tif Sample collection.

Fig. S2 msystems.00342-25-s0002.tif Bioinformatics pipeline.

Fig. S3 msystems.00342-25-s0003.tif Shellfish virus distribution patterns.

Fig. S4 msystems.00342-25-s0004.tif vOTU and NCBI refseq viral genome networks.

Fig. S5 msystems.00342-25-s0005.tif SVD distribution patterns.

Fig. S6 msystems.00342-25-s0006.tif Smacovirus phylogeny.

Fig. S7 msystems.00342-25-s0007.tif MAG Lefse analysis.

Fig. S8 msystems.00342-25-s0008.tif VF and ARG distribution patterns.

Fig. S9 msystems.00342-25-s0009.tif Toxins and antitoxin.

Supplemental tables msystems.00342-25-s0010.xlsx Tables S1 to S4.

ASM does not own the copyrights to Supplemental Material that may be linked to, or accessed through, an article. The authors have granted ASM a non-exclusive, world-wide license to publish the Supplemental Material files. Please contact the corresponding author directly for reuse.
