# Supplementary figures and images for: Healthy and moribund Zhikong scallops (Chlamys farreri) developed different viral communities during a mass mortality event
Source: mSystems. 2025 May 14;10(6):e00342-25. doi: 10.1128/msystems.00342-25 (PMC12172486; doi:10.1128/msystems.00342-25)

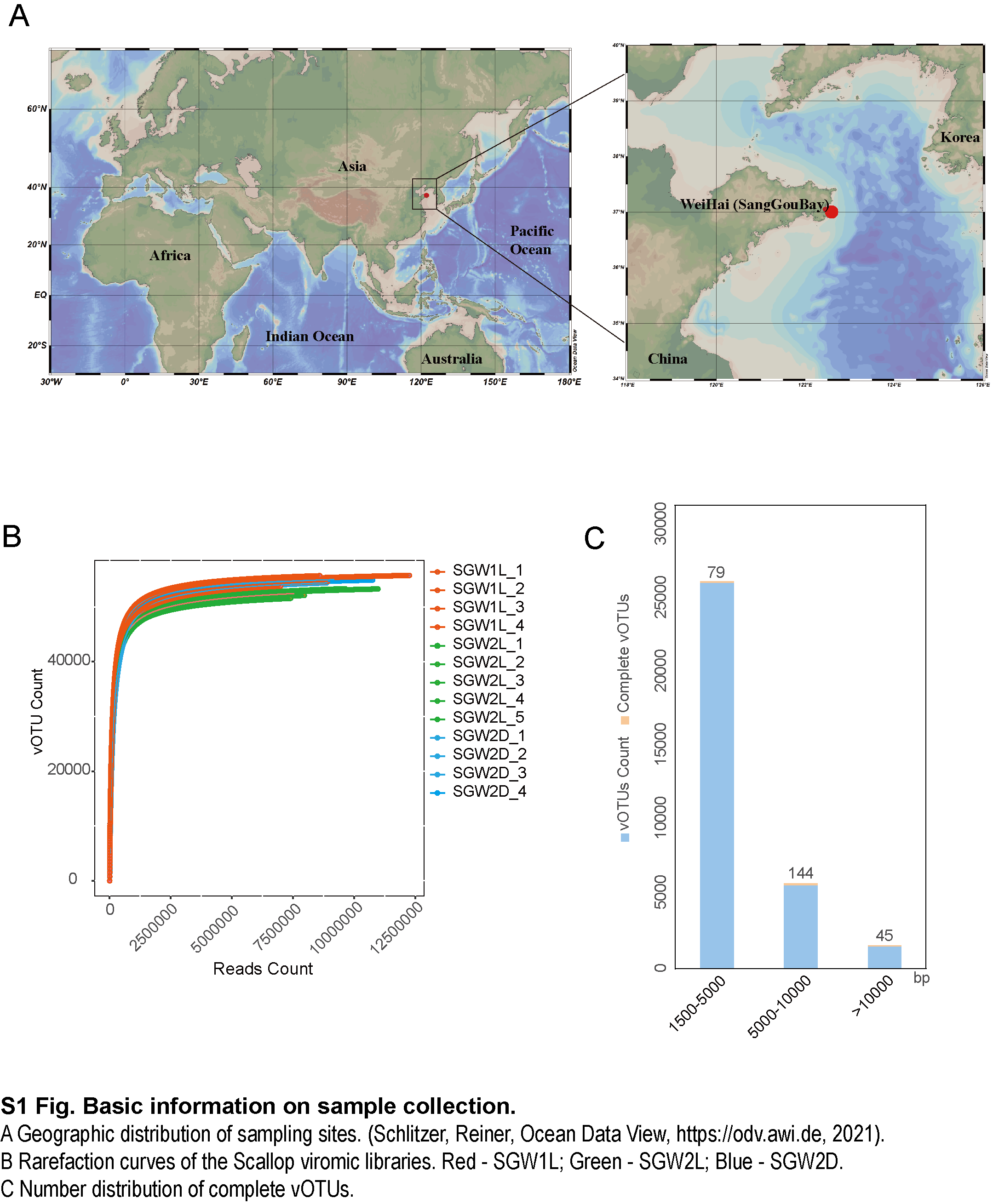

Supplement: Fig. S1 — Sample collection. [file msystems.00342-25-s0001.tif]

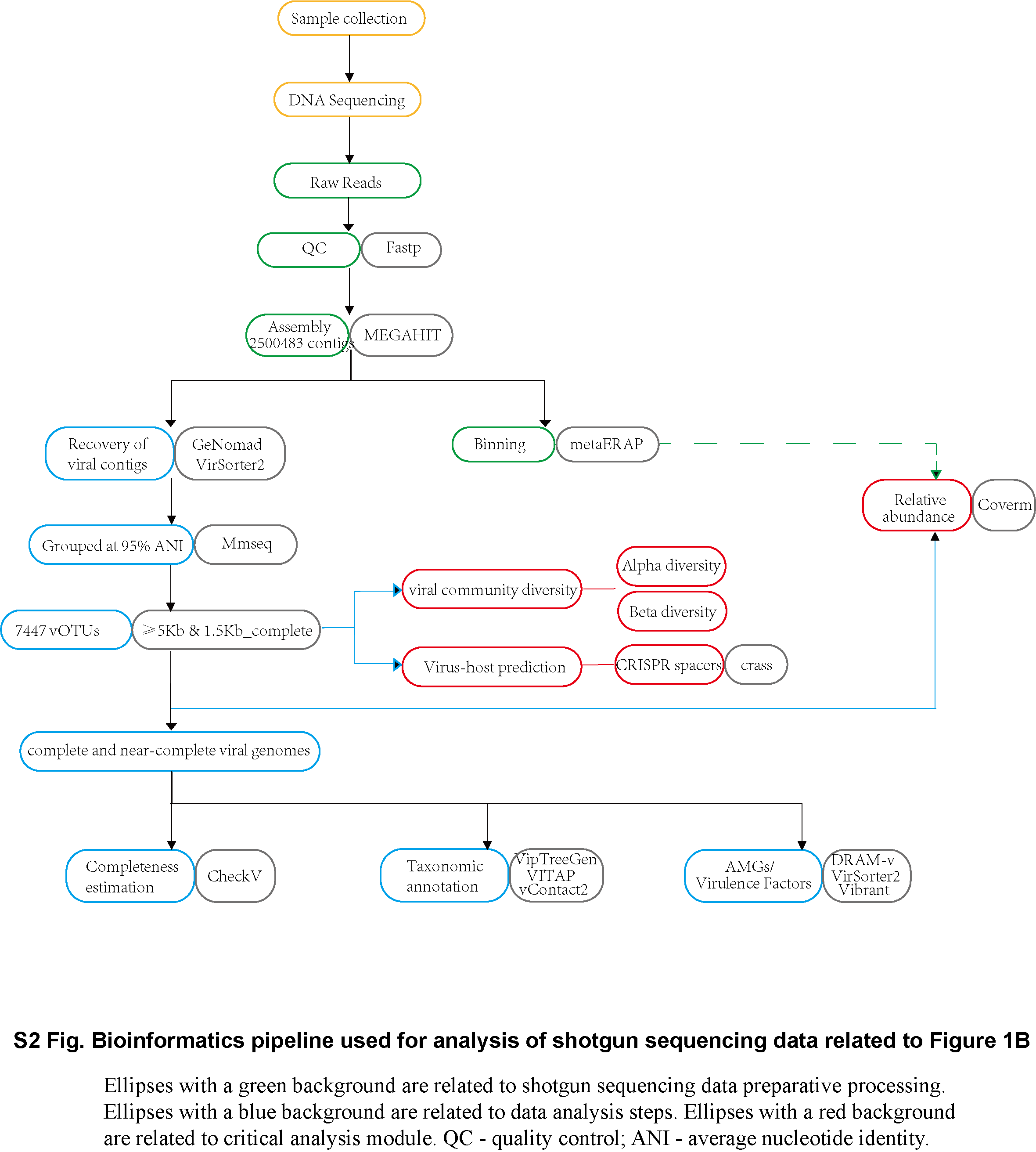

Supplement: Fig. S2 — Bioinformatics pipeline. [file msystems.00342-25-s0002.tif]

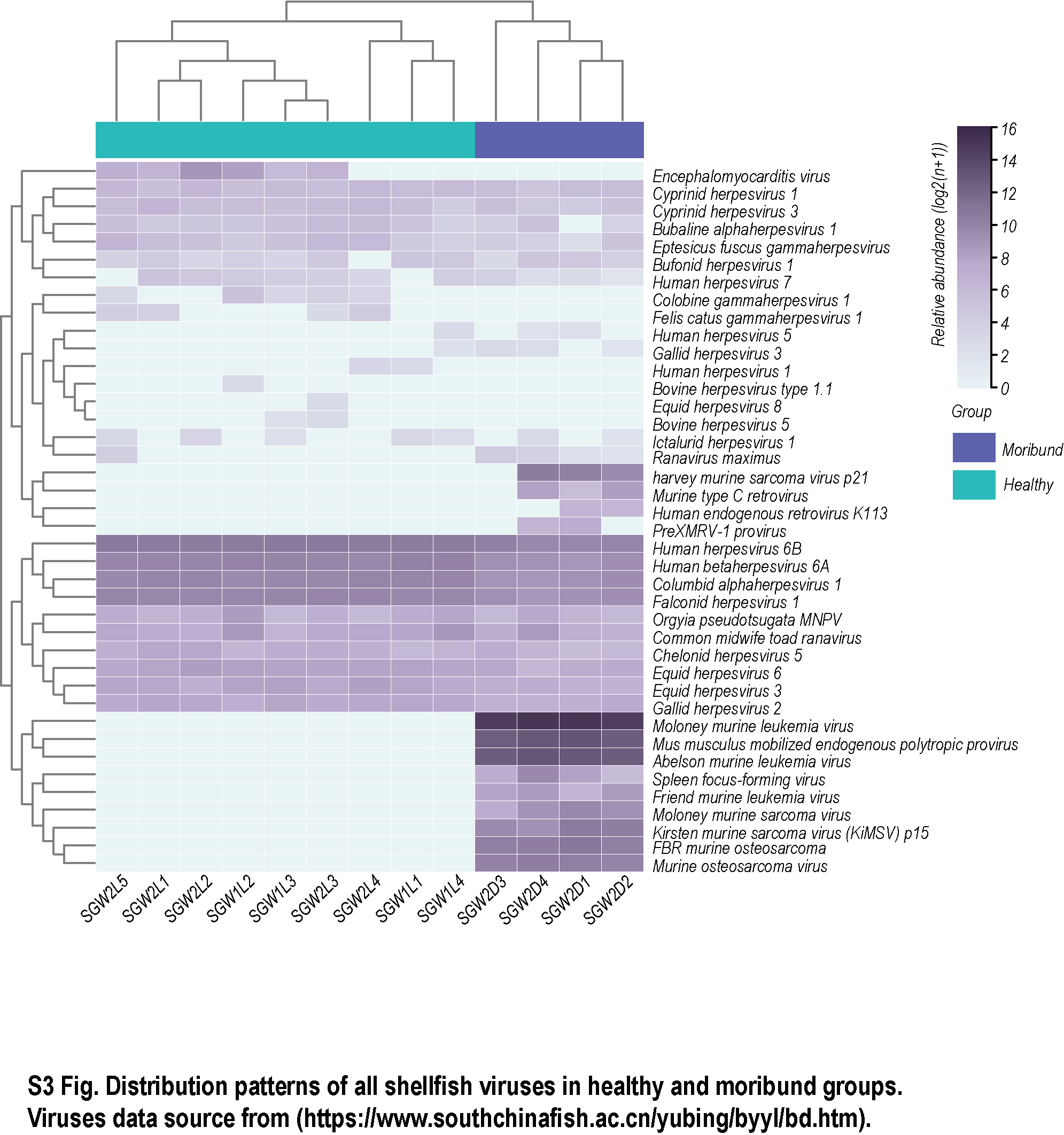

Supplement: Fig. S3 — Shellfish virus distribution patterns. [file msystems.00342-25-s0003.tif]

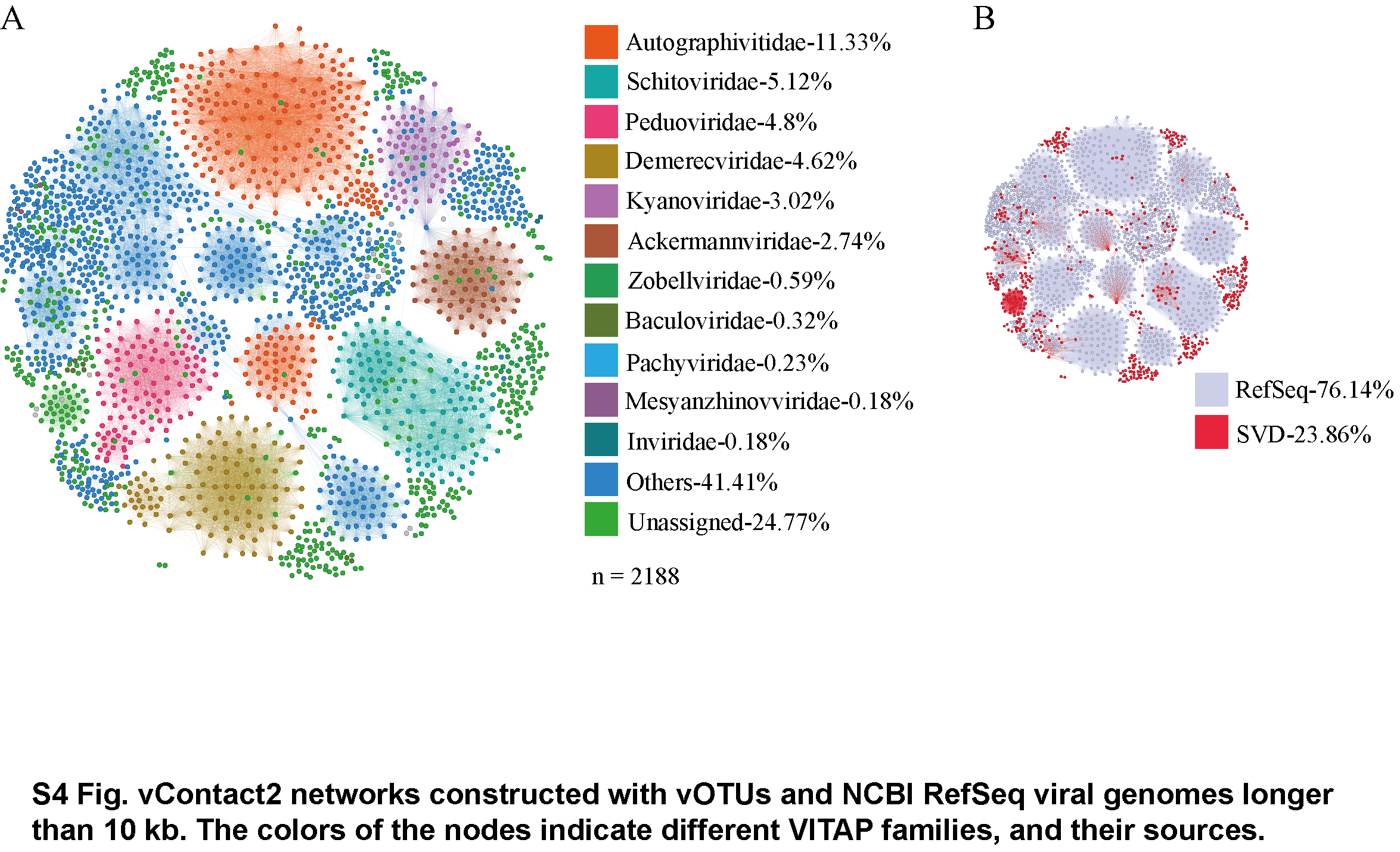

Supplement: Fig. S4 — vOTU and NCBI refseq viral genome networks. [file msystems.00342-25-s0004.tif]

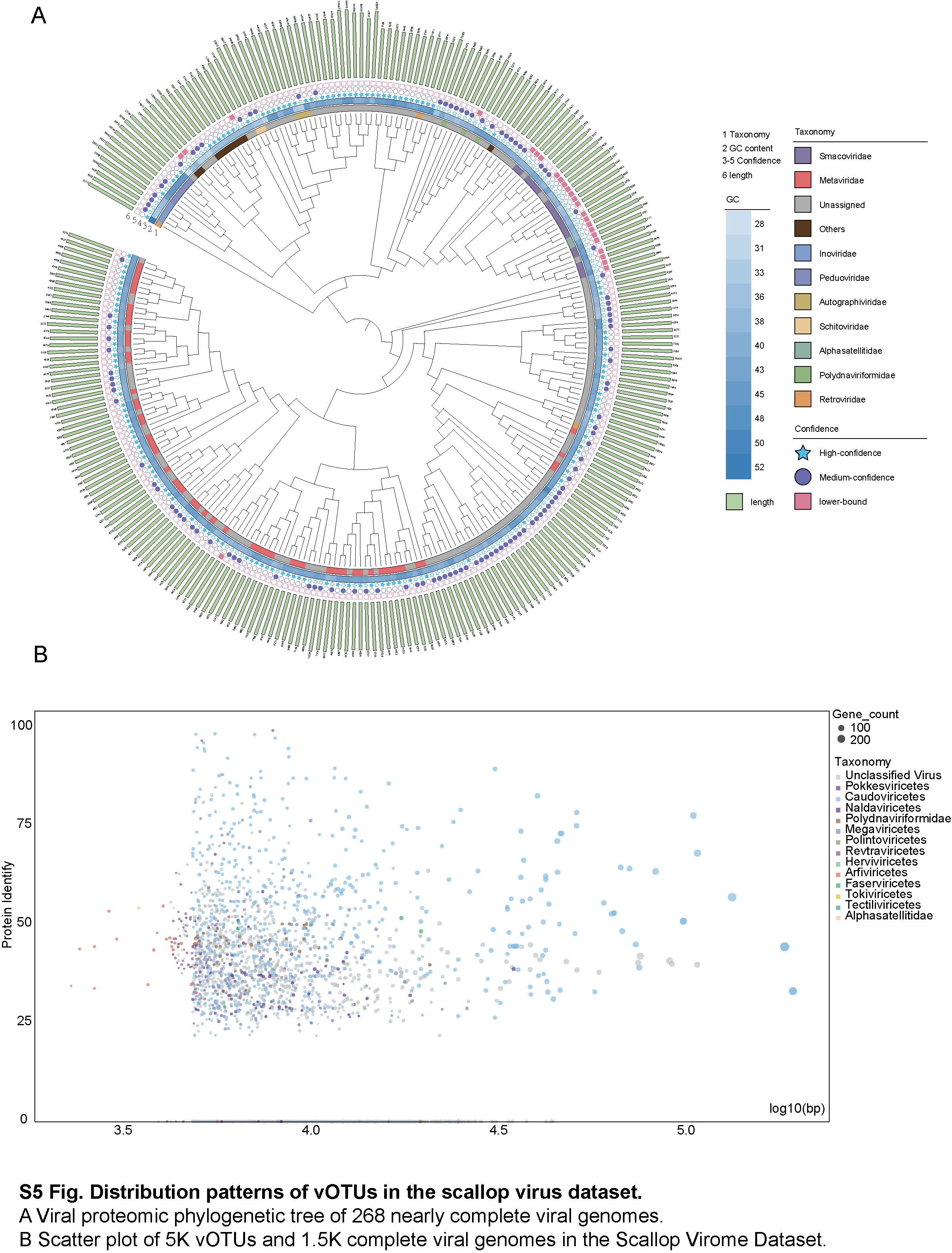

Supplement: Fig. S5 — SVD distribution patterns. [file msystems.00342-25-s0005.tif]

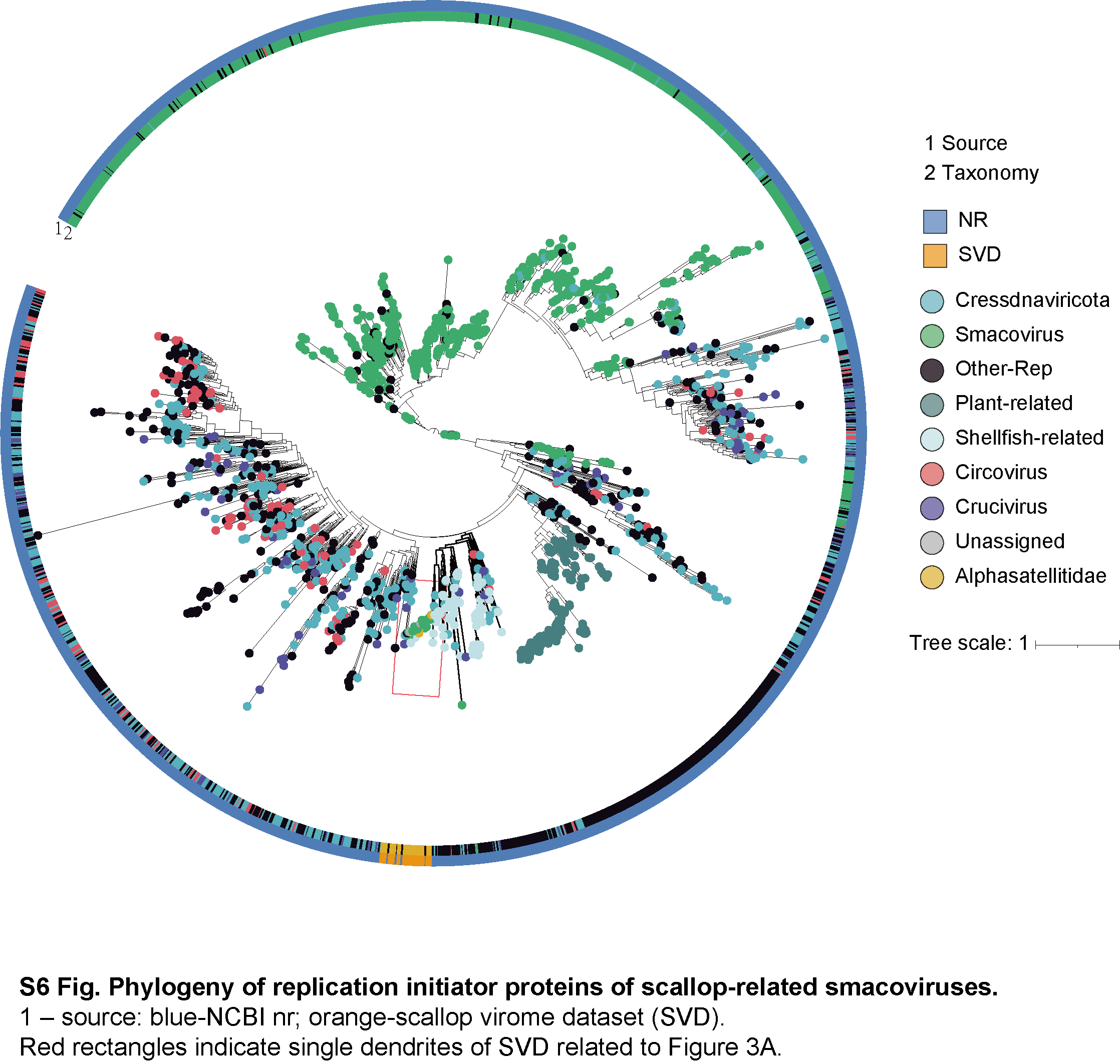

Supplement: Fig. S6 — Smacovirus phylogeny. [file msystems.00342-25-s0006.tif]

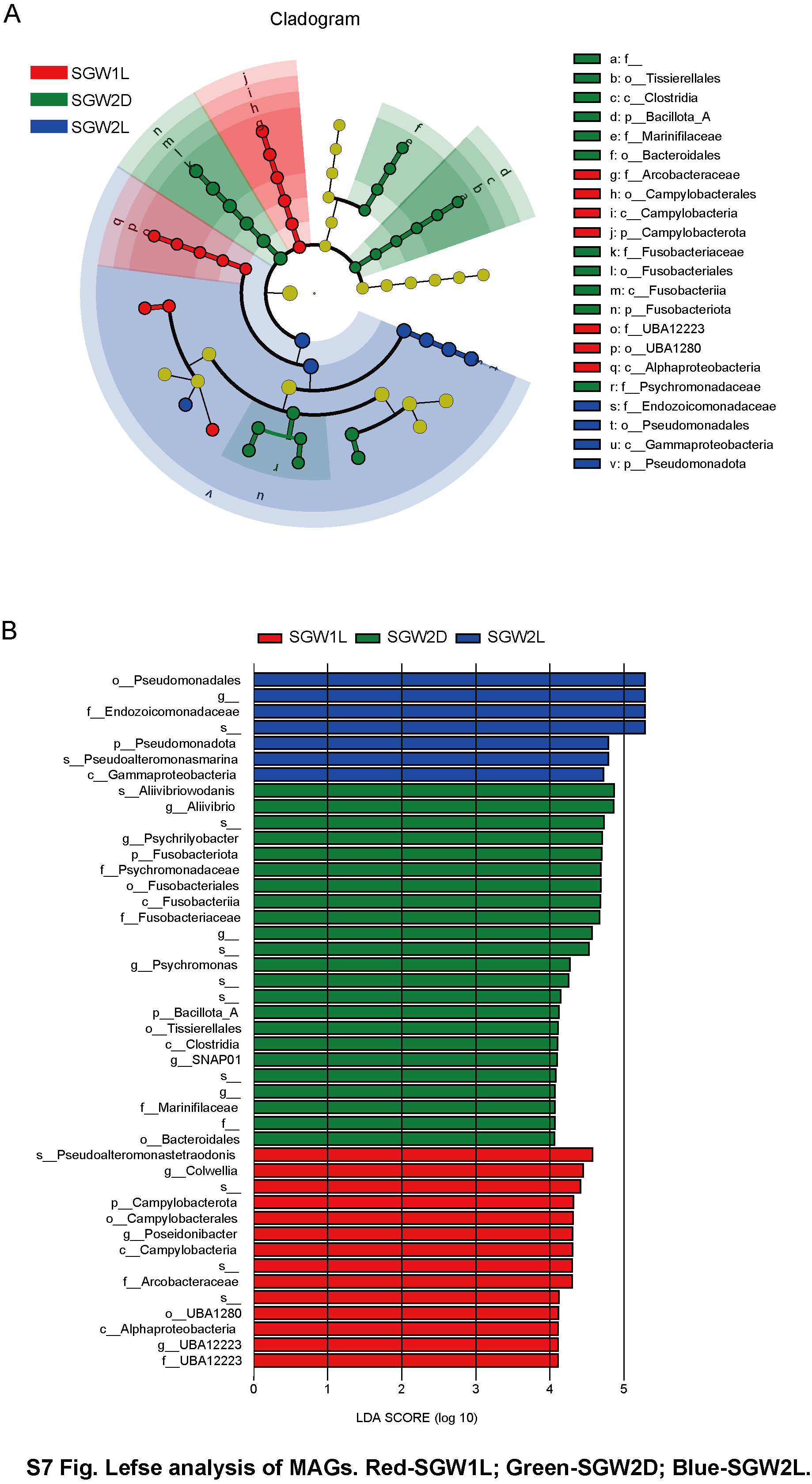

Supplement: Fig. S7 — MAG Lefse analysis. [file msystems.00342-25-s0007.tif]

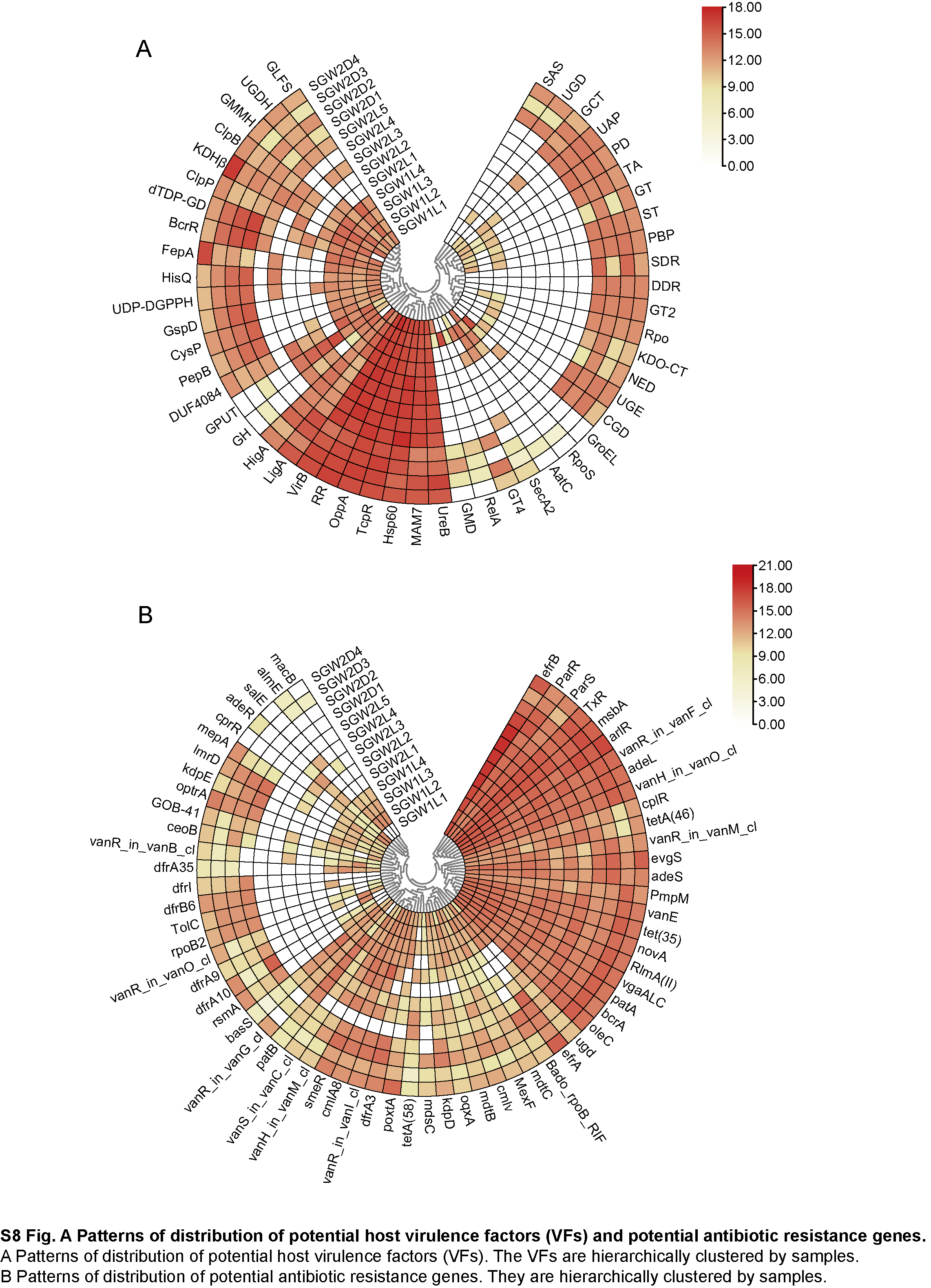

Supplement: Fig. S8 — VF and ARG distribution patterns. [file msystems.00342-25-s0008.tif]

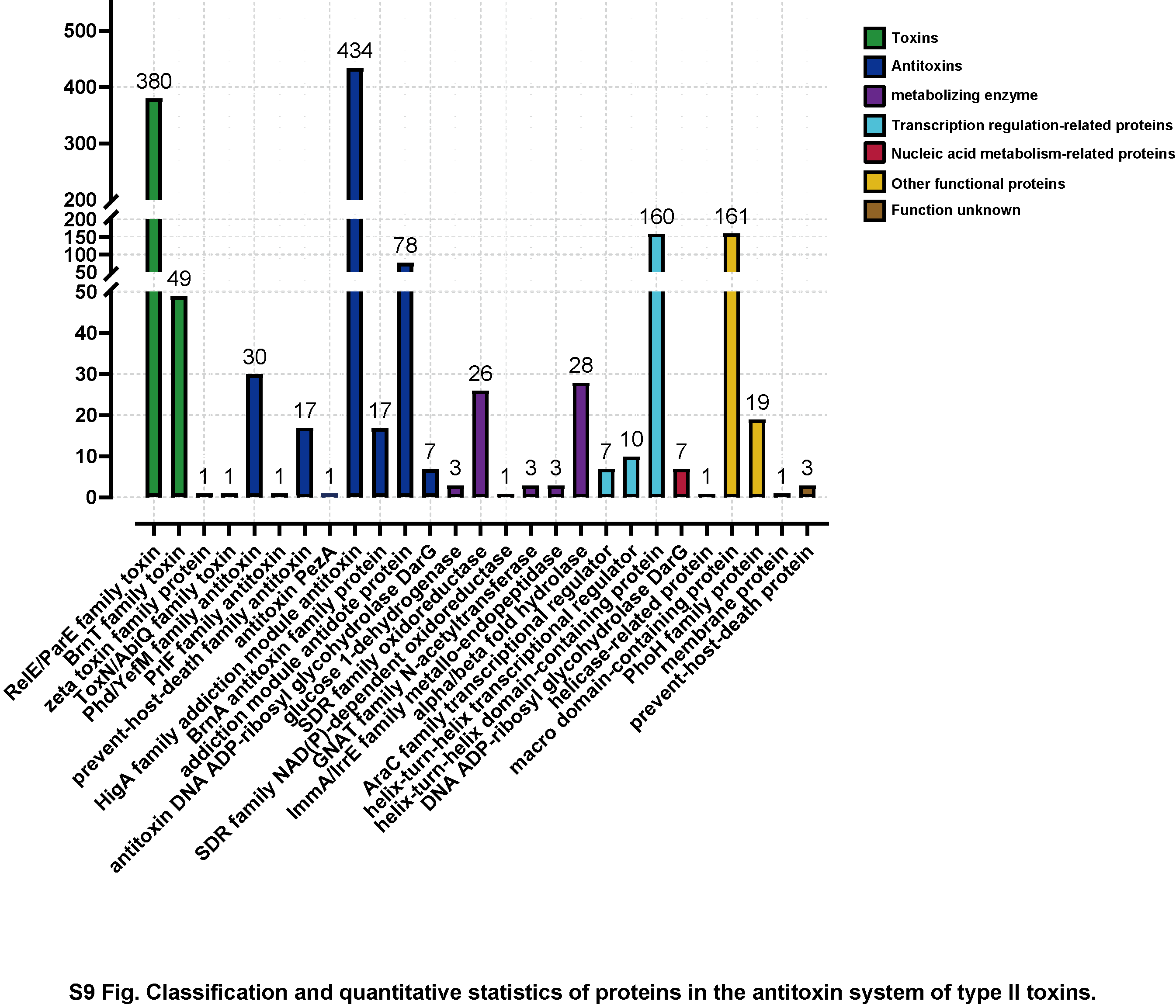

Supplement: Fig. S9 — Toxins and antitoxin. [file msystems.00342-25-s0009.tif]
